# Assisted reproductive technology (ART) patient information-seeking behavior: a qualitative study

**DOI:** 10.1186/s12905-024-03183-z

**Published:** 2024-06-15

**Authors:** Emma Mayette, Ariel Scalise, Angela Li, Nicolette McGeorge, Kaitlyn James, Shruthi Mahalingaiah

**Affiliations:** 1 Division of Reproductive Endocrinology and Infertility, Department of Obstetrics and Gynecology, Massachusetts General Hospital, 55 Fruit Street, Yawkey 10, Boston, MA 02114 USA; 2 Department of Environmental Health, Harvard T.H. Chan School of Public Health, 665 Huntington Avenue, Boston, MA 02115 USA; 3 Charles River Analytics, Inc., 625 Mount Auburn St., Cambridge, MA 02148 USA

**Keywords:** Assisted reproductive technology, In vitro fertilization, Infertility, Patient information-seeking behavior, Thematic analysis, Qualitative research

## Abstract

**Background:**

Approximately 13% of women in the United States of reproductive age seek infertility services. Assisted reproductive technology (ART), including in vitro fertilization, is used to help patients achieve pregnancy. Many people are not familiar with these treatments prior to becoming patients and possess knowledge gaps about care.

**Methods:**

This study employed qualitative methods to investigate how patients interact with information sources during care. Patients who underwent ART including embryo transfer between January 2017 and April 2022 at a large urban healthcare center were eligible. Semi-structured, in-depth interviews were conducted between August and October 2022. Fifteen females with an average age of 39 years participated. Reflexive thematic analysis was performed.

**Results:**

Two main themes emerged. Participants (1) utilized clinic-provided information and then turned to outside sources to fill knowledge gaps; (2) struggled to learn about costs, insurance, and mental health resources to support care. Participants preferred clinic-provided resources and then utilized academic sources, the internet, and social media when they had unfulfilled information needs. Knowledge gaps related to cost, insurance, and mental health support were reported.

**Conclusion:**

ART clinics can consider providing more information about cost, insurance, and mental health support to patients.

**Trial registration:**

The Massachusetts General Hospital Institutional Review Board approved this study (#2022P000474) and informed consent was obtained from each participant.

**Supplementary Information:**

The online version contains supplementary material available at 10.1186/s12905-024-03183-z.

## Background

Approximately 13% of women in the United States of reproductive age seek infertility services [1]. Treatment options include ovulation-stimulating medication, artificial insemination, and assisted reproductive technology (ART), which includes in vitro fertilization (IVF) [2]. IVF involves fertilization of extracted eggs with sperm in the laboratory and transfer of the fertilized embryo to the uterus [3]. This treatment can result in pregnancy and a live birth if successful.

Many people are unfamiliar with infertility healthcare before becoming patients [4]. Patients often possess large knowledge gaps about treatment and may have difficulty understanding ART care [5]. Patients receiving infertility treatment have reported seeking additional information on the internet to supplement their knowledge [6, 7]. In one study, 87.8% of respondents turned to the internet for additional infertility information, and 29.1% reported that they still did not find the answers they were looking for after searching online [8]. It is imperative to supply infertility patients with sufficient information resources so they can make informed decisions about care [9].

Qualitative research can answer questions of clinical relevance by providing a thorough account of patient experiences [10]. In this study, semi-structured interviews were used to investigate patient experiences during ART care. The goal of this analysis was to explore patient interaction with information resources during treatment. We have identified topics patients struggled to understand, and how they engaged with various sources to improve knowledge.

There are few existing studies that qualitatively assess patient information-seeking behavior during ART care in the United States. Investigating information resource utilization is an important first step towards supporting patient understanding of ART treatment.

## Methods

### Design

The Assisted Reproductive Technology Patient and Provider Resource to Improve Communication about Outcomes and Treatment (APRICOT) study was conducted to investigate how patients report birth outcomes to their ART clinics after becoming pregnant and moving on to receive care from an obstetrician. The aim of the APRICOT study was to understand the reporting processes for clinicians and patients by employing quantitative and qualitative methods. Participants were administered a survey that confirmed their eligibility and collected sociodemographic information, details about their infertility care, and how they reported birth outcomes to their ART clinic. At the end of the survey, participants were asked if they were interested in being interviewed about their ART experience. Participants responding “yes” were brought to a factsheet about the interview and were able to submit their contact information in the interview interest form. Interview participants were asked a variety of questions related to ART treatment, including the ways they obtained and utilized information during treatment. The qualitative data collected from interview participants about their information-seeking behavior is reported in this analysis.

Qualitative data collection was guided by a human factors engineering-based approach, which aims to assess and optimize the interaction between humans and elements of their environment [11]. This approach was selected to uncover the experiences of infertility patients with all aspects of their care including communication, information accessibility, and decision-making processes.

### Participants

Participants were identified from patient records at a large urban healthcare system. Patients were eligible for the study if they underwent ART treatment between July 2017 and April 2022. Patients were required to have undergone any form of embryo transfer, be at least eighteen years old, and be able to read and understand English. The study participants were selected through purposeful sampling of patients that underwent ART including embryo transfer [12]. Eligible patients were invited to participate in the APRICOT study which included both a survey and an interview. Participants who completed the survey and the interview interest form were contacted via phone or email to participate. Participants were selected for the interview in the order that they completed the survey, with focus placed on recruiting a sample representative of the racial and ethnic distribution of the population of Massachusetts [13]. Participant demographics were collected in the APRICOT survey prior to the interview. $50 Target gift cards were provided as compensation for completing the interview.

### Data collection

Semi-structured interviews were used to conduct an in-depth exploration of participant experiences during ART care [14]. The interview guide contained open-ended questions and participants were asked probing questions about topics they introduced [15]. The semi-structured guide was updated throughout the interview period to enhance clarity and ensure consistent probing. The complete interview guide is included in Supplementary Document 1. Video interviews via the healthcare system’s Health Insurance Portability and Accountability Act (HIPAA) compliant Microsoft Teams (*n* = 12) and telephone interviews (*n* = 3) were conducted by an undergraduate research assistant with support from a PhD-level scientist trained in qualitative methods and human factors engineering between August 2022 and October 2022. The primary interviewer was trained in qualitative techniques by a second researcher with experience conducting qualitative interviews. The interviewer did not have prior experience with ART patients.

Interview participants were provided with a study fact sheet and informed consent was obtained at the beginning of each interview. Participants consented to participation and audio recording of the interview. Interview audio was recorded using a digital voice recorder for all interviews and transcribed verbatim via a HIPAA-compliant professional transcription service. The audio files were deleted after transcription was completed. The interviews ranged from 41 to 60 min in length with a mean length of 50 min. Data saturation was reached once fifteen participants were interviewed; at this point no new ideas were brought up by participants in response to interview guide questions. The sample size was determined to be suitable because a rich data set was obtained through in-depth interviews with a homogenous population [16–18].

### Data analysis

Prior to conducting analysis, all personal identifying information was removed from the transcripts. An inductive approach was used to identify interview themes, meaning the analysis was done without applying an a priori hypothesis [19]. Thematic analysis was performed according to the six phases developed by Braun and Clarke: familiarizing yourself with your data, generating initial codes, searching for themes, reviewing themes, defining and naming themes, and producing the report [20]. Researchers familiarized themselves with the data by reading the interview transcripts. Initial code generation was performed by two researchers who each individually analyzed one interview using NVivo 12.0 software [21]. At the time of analysis, one researcher was an undergraduate research assistant and the second was a resident physician in obstetrics and gynecology and a master of public health student. After reviewing an interview transcript, researchers met to compare and combine their coding until they reached agreement for five interviews. A third researcher contributed to the decision-making process when disagreement occurred. A codebook containing the list of codes and definitions was created. The two coders then individually analyzed the remaining ten interviews, with each researcher completing five interviews. Once coding was complete, the two researchers reviewed the coding performed by the other researcher to ensure consistency. To begin searching for themes, codes related to patient information-seeking behavior were sorted into preliminary groups to organize the data. Preliminary themes containing robust support across the fifteen interviews were refined to ensure they contained concise ideas. The data extracts associated with each theme were reviewed to ensure they formed meaningful patterns that accurately represented the dataset. Researchers then reviewed compiled themes before defining and naming final themes. Participant demographic information was analyzed to determine median age at time of interview.

## Results

Fifteen female ART patients were interviewed. Interview participants received care at two different hospital sites. The median age of participants at the time of interview was 39 years. Most participants had a live birth after treatment (73.3%). Nearly all participants completed college or held post-graduate degrees (93.3%). Demographic information of interview participants is detailed in Table 1.

**Table 1:** Participant demographics

Characteristic	Median (SD)
*Age in years*	39 (0.50)
	N (%)
*Biological sex*	
Female	15 (100%)
*Race/ethnicity*	
White	8 (53.3%)
Hispanic, Latina, or Spanish Origin	2 (13.3%)
Black, African American, or African	1 (6.7%)
Southeast Asian	1 (6.7%)
South Asian	1 (6.7%)
Some other race, ethnicity, or origin	1 (6.7%)
*Education*	
Some college	1 (6.7%)
College graduate	5 (33.3%)
Postgraduate (for example masters, professional, doctorate)	9 (60%)
*Outcome of most recent ART cycle*	
Live birth	11 (73.3%)
Spontaneous abortion	1 (6.7%)
Outcome unknown	3 (20.0%)

Two main themes were identified. Interview participants: (1) utilized clinic-provided information and then turned to outside sources to fill knowledge gaps; (2) struggled to learn about costs, insurance, and mental health resources.

Theme 1: Participants utilized clinic-provided information and then turned to outside sources to fill knowledge gaps.

Participants reported using four categories of resources: clinic materials, academic sources, general internet searches, and social media. Participants reported a preference for information from their clinic and provider. They supplemented their knowledge with outside sources such as academic websites. Lastly, they reported searching the internet and social media for further information they could more easily understand.

Participants discussed feeling content with their medical care when they fully understood their treatment. They utilized outside information sources to support the knowledge gained from their provider: *“I mostly just let the clinic tell me what the protocol was going to be, and then I kind of read a little bit more about it to make sure that it made sense for me. I think I ultimately just agreed with what they decided anyways, but at least it made me feel like … I could have at least gone back and asked why they chose that or why not something else and had the conversation.” – Participant 9.* Participants felt confident in the treatment plan created by their provider after they supplemented their knowledge with additional resources.

A. Clinic materials

Participants spoke positively about the quality of information they received from their clinic and that they engaged with these sources prior to searching on their own. Participants reported a preference for receiving information from their providers:

*“I don’t like to search on my own, I’d rather just talk to a doctor.” – Participant 12*

Participants reported using clinic-provided materials; most notably they watched videos about how to administer medication:

*“I loved how they had the videos on how to do the medication, and those were up whenever you could watch them.” – Participant 1*

B. Academic sources

When participants wanted to find more information, they reported turning to sources such as academic organizations and research studies. However, they had difficulty understanding the information provided by these sources:

*“I was checking everywhere and anywhere. College resource pages…had some papers on stuff. But I don’t feel like it made me any smarter about what I was doing.****”****– Participant 10*.

C. Internet searches

Next, participants reported performing general internet searches to find answers:

*“I did google things, probably a little more than I should have, and I think that can lead you down paths that are not helpful”– Participant 13*.

Participants reported awareness that internet resources can lead to misinformation and heighten stress:

*“I actually ended up researching on the internet and all of that, there were a lot of negative things around how people’s experiences were. That scared me a lot… I thought, ‘Okay. I’m not going back– I’m not going to research.’” – Participant 14*.

D. Social media

Participants utilized social media sites to connect with other infertility patients and find more information about care:

*“So after I saw the doctor, I joined a Facebook group, and I think I learned a lot, actually, through that, just from reading other people’s questions and hearing other people’s stories, and posting my own questions on there. So I think most of the information I actually got was through that” – Participant 7*.

Participants utilized these sources to learn more about treatment, particularly anecdotal information:

*“For example, on the patient education video, they told you to put one thing in your thigh, but when I did that, I would keep hitting veins, and I had huge bruises. So then I looked online, and they all recommended a different spot in your stomach. So there were just suggestions in terms of the gauge of the needle, but that wasn’t covered in the education stuff. That was really helpful. And putting a cold compress on to numb it. That sort of thing.” – Participant 3*.

Theme 2: participants struggled to learn about ART costs, insurance, and mental health resources

Participants described being unfamiliar with ART before beginning treatment: *“I knew nothing about IVF. Nothing. Nothing. I knew that it was a possibility, but going into it, I went into it blindly.” – Participant 1.* Participants utilized various sources to learn more about ART care as they underwent treatment. They reported difficulty obtaining information related to cost, insurance, and mental health support options.

A. Costs and insurance

Many participants expressed that they were concerned about how insurance coverage would apply to their ART care:

*“I was worried about… the financial component to it, kind of wanting to know how insurance coverage works with that and how it would impact our scenario.” – Participant 15*.

Participants reported being unaware of ART costs and struggling to find financial information:

*“It was more so me trying to find out like, ‘Do I need 10 grand to start this process?’ And I couldn’t get an answer very easily.” – Participant 12*.

Participant lack of knowledge about ART costs and insurance coverage impacted their ability to plan for treatment:

*“Oh, my gosh. Just figuring out how many cycles. So my husband’s insurance only covered, I think, it was two IVF cycles and $10,000 worth of medication. I didn’t know that, basically, one cycle is like $11,000 or $11,500 in medications, so that freaked me out.” – Participant 1*.

B. Mental health resources

Many interview participants discussed high levels of emotional strain while they were undergoing ART treatment:

*“I think the whole process is just emotionally gut-wrenching. You’re kind of a basket case the whole time.” – Participant 2*.

Participants reported that they were not aware of mental health support options available to them:

*“But at the time, I don’t remember being told, “Oh, here’s someone you can talk to that can help you with this, … dealing with the mental health aspect of going through a process like this.” I don’t remember anyone ever offering or talking to me about that sort of thing, so I didn’t seek it out at the time. But looking back, obviously, I wish that I had.” – Participant 2*.

Participants wished they had sought mental health support due to the strenuous nature of treatment, but had difficulty identifying support options while receiving ART care:

*“I probably should’ve seen some kind of mental health– maybe just a counselor or a therapist, just to check in and see how I was doing, just because those were really, really dark times for me… Unfortunately, it wasn’t something easy to find.” – Participant 11*.

## Discussion

This study assessed the information-seeking experiences of infertility patients through a qualitative, patient-centered lens. Participants indicated that they value informed decision-making, prefer clinic-provided materials, and desire more information regarding cost, insurance, and mental health support tools. As noted in other literature, healthcare patients value being informed about their care so that they feel confident about their treatment plan [22]. Interview participants reported the importance of informed decision-making, reiterating the significance of this analysis to identify the types of information ART patients currently lack.

In this study, participants reported a preference for receiving information from their provider and clinic. They supplemented this knowledge with outside sources, including academic websites, research studies, internet searches, and social media. These findings are consistent with a previous study in which patients reported searching the internet for information about care but valued their physician’s opinion over outside sources [23].

Utilizing social media for health information has been observed in patients with unfulfilled information needs [24]. Obstetrics and gynecology patients have reported using social media for education, social support, and sharing of advice, but were aware that these sources can contain inaccurate information [25]. Social media can be a beneficial tool for healthcare patients to share advice and receive community support [26]. While social media enables connection with others undergoing similar experiences with infertility care, it is not a trusted tool for reliable information about treatment [27]. There is a large amount of misinformation on general internet websites, including social media websites, which can be detrimental to accurate patient knowledge about health [28]. Providing patients with validated and easily accessible information can help prevent misinformation from unregulated sources, as well as decrease the amount of time providers spend educating patients.

The desire for more information about insurance and cost of treatment is true for patients across healthcare disciplines in the United States [29]. It is expected that individuals will have difficulty locating and navigating insurance policy information [30]. Infertility coverage is difficult to navigate because of varying insurance laws and regulations in different states [31]. The need for accurate insurance and cost information is imperative as insurance coverage for ART varies widely across and within states [32]. While insurance coverage is different for every patient, infertility clinics should provide guidelines on obtaining this information due to reported complexities in this area. Providing patients with cost estimates of infertility treatments prior to care can improve their ability to make informed decisions [33].

We found that interview participants experienced high levels of emotional distress during infertility care and had difficulty identifying mental health support options. Infertility patient struggles with mental health and the limited provision of information regarding psychological support tools has been previously noted [34]. In one survey, infertility patients responded that they were not adequately informed about social support options during infertility care [35]. Meeting the psychosocial needs of infertility patients is an important aspect of patient-centered care [36]. Given the high rates of depression and anxiety experienced by patients undergoing ART [37], providers should ensure that patients are aware of mental health resources available to them, as well as the potential benefits of their utilization.

This study has several limitations. Most participants had a live birth, which may have influenced willingness to participate in the study and perception of ART treatment. Nearly all participants had completed college or professional degrees, which is not representative of the United States population. The participants received care in a state that mandates insurance coverage for ART care, but there are many states throughout the U.S. that do not ensure coverage [38]. The study sample includes patients from a single health care system that has extensive clinical resources. Therefore, the knowledge gaps reported in this study could potentially be further exacerbated at clinics with fewer resources. The sample size of fifteen participants allowed for in-depth exploration of information-seeking behavior during care. Our qualitative approach provides a rich account of how participants utilized information sources during treatment. However, the experiences of fifteen individuals may not be applicable to all ART patients. Future studies may consider investigating the knowledge gaps of a larger and more diverse cohort.

Our findings can be used by clinicians to understand the ways patients search for information during treatment, and which topics they require more information on. Providing patients with additional information resources can prevent the spread of misinformation from online sources and reduce time spent educating patients. Meeting patient information needs regarding ART costs and insurance can help improve finance-related stress. Connecting patients with mental health support tools can alleviate emotional distress during treatment. Clinicians can utilize these findings to improve patient access to reliable information sources related to ART treatment costs, insurance, and mental health support options.

ART technology is rapidly evolving and education resources must be updated to keep patients informed about their infertility treatment [39]. ART treatment, laws, and regulations can vary greatly between countries based on cultural and economic factors [40]. The results from this study can be used to inform clinics in the United States about patient interaction with information sources to learn more about ART treatment and existing patient knowledge gaps.

## Conclusion

This study qualitatively assessed the information-seeking experience of patients undergoing ART treatment. Participants reported a preference for utilizing clinic-provided information to learn about care. They next turned to academic sources, which they had difficulty understanding. Lastly, they utilized the internet and social media sites to gain more information. Participants had difficulty learning about ART costs, insurance, and support for mental health during treatment. ART clinics can consider providing patients with more information related to cost, insurance, and mental health support tools.

### Electronic supplementary material

Below is the link to the electronic supplementary material.

Supplementary Material 1

## Data Availability

De-identified data can possibly be made available upon reasonable request.

## Abbreviations

ART: Assisted Reproductive Technology
IVF: In-vitro Fertilization
HIPAA: Health Insurance Portability and Accountability Act
